# Shikonin Promotes Apoptosis and Attenuates Migration and Invasion of Human Esophageal Cancer Cells by Inhibiting Tumor Necrosis Factor Receptor-Associated Protein 1 Expression and AKT/mTOR Signaling Pathway

**DOI:** 10.1155/2021/5386050

**Published:** 2021-11-13

**Authors:** Jingrong Huang, Li Zhao, Chengxian Gong, Yi Wang, Yinzong Qu, Chunyan Ji, Jianmei Yang

**Affiliations:** Department of Gastroenterology, Hubei Provincial Hospital of Integrated Chinese & Western Medicine, Hubei, Wuhan 430015, China

## Abstract

The aim of this study was to investigate the anticancer effects of shikonin on esophageal cancer (EC) cells and explore the underlying molecular mechanism by identifying dysregulation in shikonin-induced tumor necrosis factor receptor-associated protein 1 (TRAP1) expression. The 3-(4, 5-dimethylthiazol-2-Yl)-2, 5-diphenyltetrazolium bromide assay and EDU assay were performed for cell viability determination. The reactive oxygen species level and mitochondrial membrane potential were evaluated using flow cytometry. The protein expression was detected using Western blot. In addition, cell migration and invasion were estimated. These results demonstrated that shikonin inhibited EC cell growth in a concentration-dependent manner and induced apoptosis through activation of the intracellular apoptotic signaling pathway. Moreover, TRAP1 downregulation promoted shikonin-induced reactive oxygen species release, whereas TRAP1 upregulation blocked it. Meanwhile, shikonin significantly promoted mitochondrial depolarization, accompanied by a large release of cytochrome C. Conversely, shikonin significantly decreased adenosine 5′-triphosphate release, demonstrating a significant intervention in the process of the glucose metabolism. In addition, not only shikonin but also short hairpin RNA (shRNA)-TRAP1 inhibited EC cell migration and invasion. shRNA-TRAP1 enhanced the inhibitory effect of shikonin on matrix metalloproteinase (MMP)2 and MMP9 expression. More interestingly, we demonstrated that shRNA-TRAP1 played a synergistic role in shikonin-mediated regulation of protein kinase B (AKT)/mammalian target of rapamycin (mTOR) signaling. Collectively, shikonin promoted apoptosis and attenuated migration and invasion of EC cells by inhibiting TRAP1 expression and AKT/mTOR signaling, indicating that shikonin may be a new drug for treating EC.

## 1. Introduction

Esophageal cancer (EC) is one of the most aggressive and lethal gastrointestinal malignancies in East Asia, often manifesting symptoms such as dysphagia and pain [1]. It usually occurs as either of two primary histologic types, esophageal squamous cell carcinoma (ESCC) or esophageal adenocarcinoma [2], among which the incidence of ESCC is higher [3]. Despite improvements in diagnosis and therapy, the five-year survival rate of EC patients still remains low due to the intense proliferation and invasion of EC cells [4]. Therefore, it is of great importance to elucidate the molecular mechanisms underlying EC growth and metastasis and to develop better therapeutic approaches.

Tumor necrosis factor receptor-associated protein 1 (TRAP1), an important member of the mitochondrial heat shock protein 90 family, has antiapoptotic and antioxidant properties [5]. It protects cancer cells from oxidative stress and apoptosis induced by certain antitumor agents and various stressors [6, 7]. It has been reported that TRAP1 may be relevant to drug resistance in colorectal and ovarian cancers [8, 9]. In addition, TRAP1 can regulate cell morphology, proliferation, migration, and invasion [10, 11]. The expression of TRAP1 can be considered as a candidate biomarker to predict prognosis of various cancers [12, 13]. In clinical ESCC patients, follow-up analysis revealed that high TRAP1 expression was associated with poor prognosis, suggesting that TRAP1 may be an independent prognostic factor for ESCC patients [11]. However, the specific biological mechanisms of TRAP1 have not yet been evaluated.

Shikonin, an active naphthoquinone of Zicao, is derived from the roots of the Chinese herb *Lithospermum erythrorhizon,* which has been used in traditional Chinese medicine to treat skin diseases, burns, and sore throats due to its antimicrobial and anti-inflammatory activities [14, 15]. Since chronic inflammation promotes the initiation and progression of cancer, the anti-inflammatory role of shikonin may contribute to its therapeutic activities against cancer such as inhibiting cell proliferation, migration, invasion, and metastasis and promoting apoptosis. For example, Jang et al. demonstrated that shikonin attenuated human breast cancer cell migration and invasion through suppressing the activation of matrix metalloproteinase 9 (MMP9) [16]. Furthermore, shikonin has been reported to exhibit suppressive effects on endometrial cancer cells through promoting apoptosis and blocking cell cycle progression [17]. However, the molecular mechanism underlying the anticancer effects of shikonin on EC cells remains unclear.

Herein, we hypothesized that shikonin exerts anticancer effects on human EC by modulating TRAP1 expression. We explored the underlying molecular mechanisms therein by identifying dysregulation in shikonin-induced TRAP1 expression. Our results suggested that shikonin exerts anticancer effects against EC through inhibiting TRAP protein kinase B (AKT)/mammalian target of rapamycin (mTOR) signaling pathway and is a potential therapeutic agent for the treatment of EC.

## 2. Materials and Methods

### 2.1. Chemicals and Reagents

The human EC cell line TE-1 (product code: TCHu 89) was purchased from the Cell Bank of the Chinese Academy of Sciences (Shanghai, China). Shikonin (HPLC, ≥98%) was purchased from Sigma (product code: S7576). Roswell Park Memorial Institute (RPMI)-1640 medium, fetal bovine serum (FBS), penicillin G, and streptomycin were obtained from Gibco BRL (Gaithersburg, MD, USA). Cell counting kit-8 (CCK-8, product code: C0038), Hoechst 33258 staining kit (product code: C0003), reactive oxygen species (ROS) assay kit (product code: S0033), and hypersensitive electrochemiluminescence kit (product code: P0018M) were purchased from Beyotime Biotech (Shanghai, China). Transwell chamber (product code: 353093) and BD Matrigel Basement Membrane Matrix (product code: 356234) were purchased from BD Biosciences (New Jersey, USA). Lipofectamine 2000 (product code: 11668019) was purchased from Thermo Fisher Scientific (Waltham, MA, USA). Adenosine 5′-triphosphate (ATP) content detection kit (product code: A095), total protein extraction kit (product code: W034), and total protein bicinchoninic acid (BCA) assay kit (product code: A045-3) were purchased from Nanjing Jiancheng Bioengineering Institute (Jiangsu, China). Mitochondrial membrane potential detection kit (JC-1, product code: PAB180068), rabbit primary antibodies against TRAP1 (product code: PAB33450), MMP2 (product code: PAB30618), MMP9 (product code: PAB30102), cleaved caspase-3 (product code: PAB30665), B-cell lymphoma 2 (Bcl-2, product code: PAB30041), Bcl-2-associated X protein (Bax, product code: PAB30727), AKT (product code: PAB35149), p-mTOR (product code: PAB36313-P), mTOR (product code: PAB30674), GAPDH (product code: PAB36264), and goat anti-rabbit IgG secondary antibody (product code: PAB160011) were purchased from Bioswamp (Wuhan, China). Rabbit primary antibodies against p-AKT (product code: ab81283) and cytochrome C (Cyt-C, product code: ab90529) were purchased from Abcam (Cambridge, UK). Other materials with the highest analytical grade were purchased from Sinopharm Chemical Reagent Co., Ltd. (Shanghai, China).

### 2.2. Cell Culture

TE-1 cells were cultured in the RPMI-1640 medium containing 10% FBS, 100 units/mL penicillin, and 100 mg/mL streptomycin. Cells were maintained at 37°C in an atmosphere containing 95% humidified air and 5% CO_2_ and passaged three times before they were subjected to experimentation. In all cell experiments, the culture medium was replaced with the serum-free medium for 12 h before drug administration.

### 2.3. Shikonin Treatment and CCK-8 Assay

Inhibition of cell proliferation by shikonin was measured by the CCK-8 assay according to the manufacturer's instructions. TE-1 cells at the logarithmic growth phase were seeded in 96-well plates at a density of 1 × 104 cells/well and treated with different concentrations of shikonin (0, 1, 5, and 10 *μ*M). After 24 h of incubation, 10 *μ*L of CCK-8 reagent was added to each well, followed by incubation at 37°C for an additional 3 h. The absorbance was then measured at a wavelength of 450 nm using a SpectraMax 190 microplate reader (Molecular Devices LLC, Sunnyvale, CA, USA). The cell viability was calculated by the following formula: cell viability (%) = (OD treatment group/OD untreated group × 100) %, where OD represents the optical density.

### 2.4. EDU Proliferation Assay

For 5-ethynyl-20-deoxyuridine (EDU) staining, different concentrations of shikonin (0, 1, 5, and 10 *μ*M) were cultured in 96-well plates. After 24 h of incubation, EDU markers (C10310-1; RiboBio, China) was added at a concentration of 50 *μ*t, and the plates were incubated for 2 h at 37°C. Then, cells were harvested for EDU staining according to the manufacturer's instructions. The images were observed using fluorescence microscopy (Olympus, Tokyo, Japan).

### 2.5. Cell Transfection

Short-hairpin RNA (shRNA) targeting TRAP1 (shRNA-TRAP1), TRAP1 overexpression vector pCDH-TRAP1 (Over-TRAP1), and pCDH vector were designed and synthesized by RiboBio Co., Ltd. (Guangzhou, China). The following primers were used for the amplified target fragment of TRAP1 (1488 bp): forward, 5′-GCTCTAGAATGGTGGCTGACAGAGTG-3′; reverse, 5′-CGGAATTCTCAGTGTCGCTCCAGGGC-3′. The interference target sequence of shRNA-TRAP1 was 5′-GGTTCTGGAGTGTTTGAAATC-3′. For transfection, TE-1 cells were incubated with shRNA-TRAP1, pCDH-TRAP1, or pCDH vectors using Lipofectamine 2000 according to the manufacturer's instructions, and the cells were harvested 24 h after transfection. The transfection efficiency was observed under the fluorescence microscope (Olympus, Tokyo, Japan). The experiment was divided into eight groups: control, shRNA-TRAP1, over-TRAP1, vector, shikonin, shikonin + shRNA-TRAP1, shikonin + over-TRAP1, and shikonin + vector.

### 2.6. Hoechst 33258 Staining

Apoptosis was determined using the Hoechst 33258 staining method. After 24 h of treatment, cells were fixed with 4% formaldehyde in phosphate-buffered saline (PBS) for 10 min, stained using Hoechst 33258 (10 mg/L) for 1 h, and observed under the fluorescence microscope (Olympus, Tokyo, Japan).

### 2.7. ROS Detection

The level of intracellular ROS was evaluated using a ROS assay kit. After 24 h of treatment, the cells were harvested, washed with 1 × PBS, and stained with 20 *μ*M dichlorodihydrofluorescein diacetate (DCFH-DA; 1 : 1,000) for 20 min at 37°C. The signal was read using flow cytometry (Beckman Coulter, Brea, CA, USA) after the cells were further washed three times using PBS. Cells treated with Rosup (provided by the ROS assay kit) only were used as negative controls.

### 2.8. Detection of Mitochondrial Membrane Potential

Mitochondrial membrane potential was estimated by the fluorescent dye 5,5′,6,6′-tetrachloro-1,1′,3,3′-tetraethyl-imidacarbocyanine (JC-1). After 24 h of treatment, the cells were washed with PBS and incubated with 10 *μ*M JC-1 for 30 min at 37°C in the dark. After resuspension with PBS, the cells were detected using flow cytometry (Beckman Coulter, Brea, CA, USA). The ratio between green fluorescence (B4 quadrant) and red fluorescence (B2 quadrant) indicated the level of mitochondrial depolarization.

### 2.9. Invasion and Migration Assays

To assess cell invasion, an 8 *μ*m pore polycarbonate membrane filter was inserted into each transwell chamber and coated with 50 *μ*L of Matrigel with a final concentration of 4 mg/mL. A total of 5 × 103 nontransfected or transiently transfected TE-1 cells were then seeded into the upper chamber with 100 *μ*L of serum-free medium, and 1 mL of RPMI-1640 medium containing 20% FBS was added to the bottom chamber. For cells treated with shikonin, 5 *μ*M shikonin was added to the bottom chamber along with 1 mL of RPMI-1640 medium containing 20% FBS. The cells were further incubated at 37°C for 24 h, after which the noninvading cells were removed from the upper surface of the filter membrane. The invading cells on the lower surface of the filter membrane were stained with crystal violet for 1 h, rinsed with water, and dried. Cells from three random visual fields each filter were captured using an optical microscope (Olympus, Tokyo, Japan) at 200× magnification and counted for quantification. Cell migration assay was performed similarly, but without Matrigel coating on the filter.

### 2.10. ATP Assay

Generally, a drop in the ATP level indicates impaired or decreased mitochondrial function. The drop in the ATP level during apoptosis is usually accompanied by a drop in mitochondrial membrane potential. Intracellular ATP levels were quantified using an ATP content detection kit following the standard protocol provided by the vendor. The ATP concentrations of the samples were calculated using an ATP standard and normalized to the protein concentrations of the samples, which were determined using the BCA protein assay kit.

### 2.11. Protein Extraction and Western Blot

Total proteins were extracted from cells using a total protein extraction kit and quantified by the BCA method. Whole proteins were electrophoresed in 10–15% polyacrylamide gels. The separated proteins were transferred to polyvinylidene difluoride membranes, and nonspecific binding was blocked with 5% skimmed milk for 2 h at 37°C. The membranes were then incubated at 4°C overnight with rabbit antibodies against TRAP1 (1 : 1000), cleaved caspase-3 (1 : 1000), Bax (1 : 1000), Bcl-2 (1 : 1000), Cyt-C (1 : 1000), MMP2 (1 : 1000), MMP9 (1 : 1000), p-AKT (1 : 5000), AKT (1 : 1000), p-mTOR (1 : 1000), and mTOR (1 : 1000). GAPDH (1 : 2000) was used as a control. Next, the membranes were incubated with goat anti-rabbit IgG (1 : 20000) for 1 h at25 ± 2°C. Images were acquired using a multifunctional gel imaging system (Image Quant LAS 500, General Electric, and Fairfield, CT, USA) after incubation with enhanced chemiluminescence reagent.

### 2.12. Statistical Analysis

All values were presented as the mean ± standard deviation. One-way ANOVA was performed using SPSS 19.0 software, followed by Turkey's post hoc test to compare differences between multiple groups (more than two) and *t*-test between two groups (IBM Corp., Armonk, NY, USA). *P* < 0.05 was considered statistically significant.

## 3. Results

### 3.1. Shikonin Showed Antiproliferation Activity and shRNA-TRAP1 Enhanced Shikonin-Induced Cell Apoptosis

**Figure 1:**
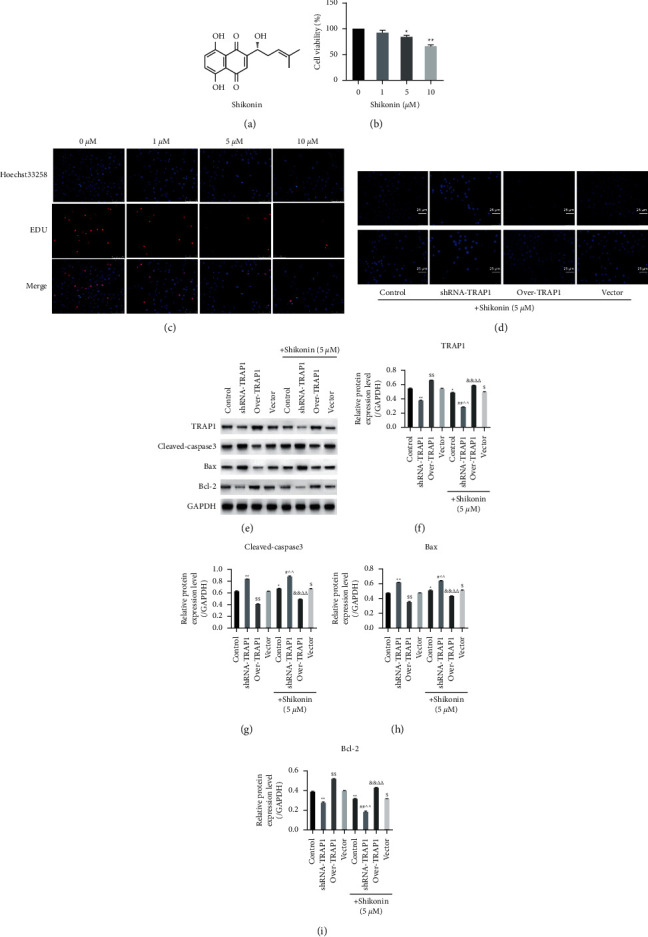
Shikonin showing antiproliferation activity and shRNA-TRAP1 enhancing shikonin-induced apoptosis in TE-1 cells. (a) Chemical structural formula of shikonin. (b) Comparison of cell viability (%);  ^*∗∗*^*P* < 0.05,  ^*∗∗*^*P* < 0.01 vs. 0 *μ*M shikonin. (c) Cell proliferation was investigated using the EDU assay, scale bar = 100 *μ*. (d) Apoptosis assay by Hoechst 33258 staining, scale bar = 25 *μ*m; (e) Western blot of apoptosis-related proteins (cleaved caspase-3, Bax, and Bcl-2) and TRAP1. (f)–(i) Quantification of each indicator. Over indicates overexpression. ^*∗*^*P* < 0.05,  ^*∗∗*^*P* < 0.01 vs. control;  ^$^*P* < 0.05, ^$$^*P* < 0.01 vs. vector; ^#^*P* < 0.05, ^##^*P* < 0.01 vs. shRAN-TRAP1; ^&&^*P* < 0.01 vs. over-TRAP1; ^∧∧^*P* < 0.01 vs. shikonin; ^ΔΔ^*P* < 0.01 vs. shikonin + vector.

Figure 1(a) shows the chemical structure of shikonin. We evaluated changes in cell proliferation by calculating cell viability relative to untreated (control) cells. The viability of TE-1 cells treated with 5 *μ*M or 10 *μ*M shikonin was significantly inhibited (*P* < 0.05 and *P* < 0.01, respectively) compared with that of untreated cells (Figure 1(b)), demonstrating the antiproliferative effect of shikonin. Furthermore, EDU analysis showed that the EDU-positive cells were lower treated with 5 *μ*M or 10 *μ*M shikonin (Figure 1(c)). In subsequent treatments, 5 *μ*M was selected as the concentration of shikonin.

With or without shikonin treatment, shRNA-TRAP1 promoted TE-1 cell apoptosis compared with that of untreated cells, while over-TRAP1 inhibited apoptosis compared with that of the nontransfected (vector) cells. However, compared with cells that were not treated by shikonin, the corresponding shikonin-treated cells showed increased apoptosis to different extents (Figure 1(c)). In addition, we examined the expression of apoptosis-related proteins (cleaved caspase-3, Bax, and Bcl-2) and TRAP1 (Figure 1(d)). Compared with the untreated group, shRNA-TRAP1 or shikonin upregulated the expression of cleaved caspase-3 and Bax (Figures 1(f) and 1(g), *P* < 0.05 and *P* < 0.01, respectively) and downregulated that of TRAP1 and Bcl-2 (Figures 1(e) and 1(h), *P* < 0.05 and *P* < 0.01, respectively). Compared with the vector group, over-TRAP1 upregulated the expression of TRAP1 and Bcl-2 (Figures 1(e) and 1(h), *P* < 0.01) and downregulated that of cleaved caspase-3 and Bax (Figures 1(f) and 1(g), *P* < 0.01), whereas shikonin exerted the opposite effect. The combination of shRNA-TRAP1 and shikonin synergistically affected each indicator, whereas the effect of over-TRAP1 on apoptosis was antagonistic (Figures 1(e)–1(h)). These results demonstrated that shRNA-TRAP1 enhanced shikonin-induced apoptosis in TE-1 cells.

### 3.2. shRNA-TRAP1 Enhanced Shikonin-Induced ROS and Mitochondrial Depolarization

**Figure 2:**
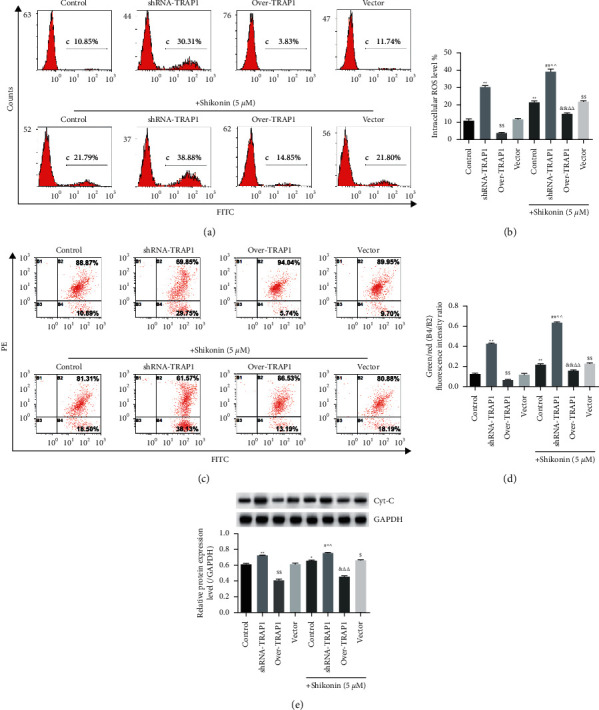
shRNA-TRAP1 enhancing shikonin-induced ROS production and mitochondrial depolarization in TE-1 cells. (a) ROS assay by flow cytometry. (b) Comparison of intracellular ROS levels. (c) Mitochondrial membrane potential assay by flow cytometry. (d) Comparison of mitochondrial membrane potential. (e) Western blot and quantification of Cyt-C. Over indicates overexpression. ^*∗*^*P* < 0.05,  ^*∗∗*^*P* < 0.01 vs. control;  ^$^*P* < 0.05, ^$$^*P* < 0.01 vs. vector; ^#^*P* < 0.05, ^##^*P* < 0.01 vs. shRAN-TRAP1; ^&^*P* < 0.05, ^&&^*P* < 0.01 vs. over-TRAP1; ^∧∧^*P* < 0.01 vs. shikonin; ^ΔΔ^*P* < 0.01 vs. shikonin + vector.

Intracellular ROS levels were increased in TE-1 cells by shRNA-TRAP1 or shikonin compared with that in the untreated cells (Figures 2(a) and 2(b), *P* < 0.01). Compared with the vector group, over-TRAP1 decreased the level of intracellular ROS (*P* < 0.01), while shikonin increased it (*P* < 0.01). The combination of shikonin and shRNA-TRAP1 synergistically increased the level of intracellular ROS compared with that of single shikonin treatment (*P* < 0.01).

The mitochondrial depolarization of cells treated by shRNA-TRAP1 or shikonin was greater than that of untreated cells (Figures 2(c) and 2(d), *P* < 0.01). Compared with the vector group, over-TRAP1 reduced mitochondrial depolarization (*P* < 0.01), while shikonin increased it (*P* < 0.01). Combined shikonin treatment also increased mitochondrial depolarization compared with shRNA-TRAP1 or over-TRAP1 (*P* < 0.01). The combination of shikonin and shRNA-TRAP1 synergistically increased mitochondrial depolarization compared with single shikonin treatment (*P* < 0.01). Western blot showed that the trend of Cyt-C protein expression was consistent with mitochondrial depolarization (Figure 2(e)). The above results demonstrated that shRNA-TRAP1 enhanced shikonin-induced intracellular ROS and mitochondrial depolarization in TE-1 cells.

### 3.3. shRNA-TRAP1 Enhanced Shikonin-Induced Inhibition of Cell Migration and Invasion

**Figure 3:**
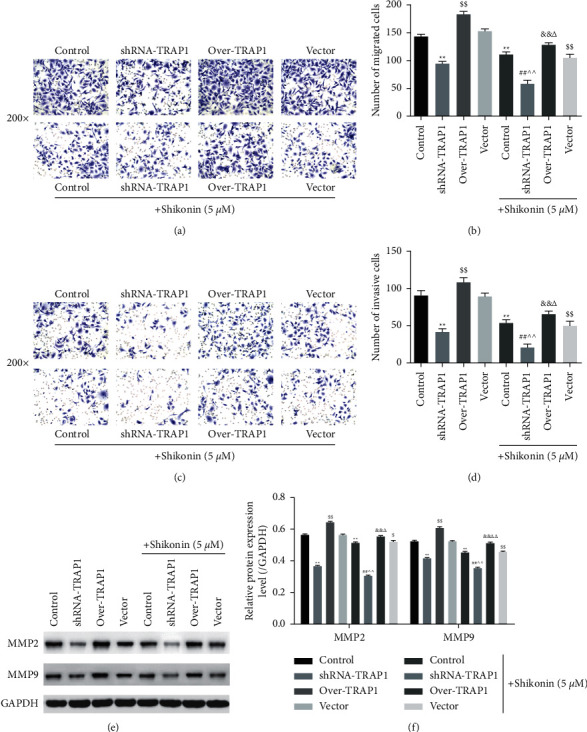
shRNA-TRAP1 enhancing shikonin-induced inhibition of migration and invasion in TE-1 cells. (a) Cell migration assay, ×200. (b) Comparison of number of migrated cells. (c) Cell invasion assay, ×200. (d) Comparison of number of invasive cells. (e) Western blot and (f) quantification of MMP2 and MMP9. Over indicates overexpression.  ^*∗∗*^*P* < 0.01 vs. control; ^$$^*P* < 0.01 vs. vector; ^##^*P* < 0.01 vs. shRAN-TRAP1; ^&^*P* < 0.05, ^&&^*P* < 0.01 vs. over-TRAP1; ^∧∧^*P* < 0.01 vs. shikonin; ^Δ^*P* < 0.05, ^ΔΔ^*P* < 0.01 vs. shikonin + vector.

We assessed changes in cell migration capacity by counting the number of migrated TE-1 cells, which was reduced with shRNA-TRAP1 or shikonin treatment compared with that of the untreated cells (Figures 3(a) and 3(b), *P* < 0.01). Compared with the vector group, over-TRAP1 increased the number of migrated TE-1 cells (*P* < 0.01), while shikonin decreased it (*P* < 0.01). Combined shikonin treatment also increased the number of migrated TE-1 cells compared with the shRNA-TRAP1 or over-TRAP1 group (*P* < 0.01). The combination of shikonin and shRNA-TRAP1 synergistically decreased the number of migrated TE-1 cells compared with single shikonin treatment (*P* < 0.01), whereas over-TRAP1 increased the number of migrated TE-1 cells (*P* < 0.05). The trend of cell invasion was similar to that of migration (Figures 3(c) and 3(d)). In addition, we examined changes in MMP2 and MMP9, which are proteins associated with cell migration and invasion (Figures 3(e) and 3(f)). Compared with the untreated cells, those treated with shRNA-TRAP1 or shikonin showed decreased expression of MMP2 and MMP9 (*P* < 0.01). Compared with the vector group, over-TRAP1 increased MMP2 and MMP9 expression (*P* < 0.01), while shikonin decreased it (*P* < 0.05, *P* < 0.01). Combined shikonin treatment also decreased the expression of MMP2 and MMP9 compared with the shRNA-TRAP1 or over-TRAP1 group (*P* < 0.01). The combination of shikonin and shRNA-TRAP1 synergistically decreased the expression of MMP2 and MMP9 compared with single shikonin treatment (*P* < 0.01), whereas over-TRAP1 showed the opposite effect (*P* < 0.05 and *P* < 0.01). These results demonstrated that shRNA-TRAP1 enhanced shikonin-induced inhibition of TE-1 cell migration and invasion.

### 3.4. shRNA-TRAP1 Enhanced the Inhibitory Effect of Shikonin on ATP Release and AKT/mTOR Signaling

**Figure 4:**
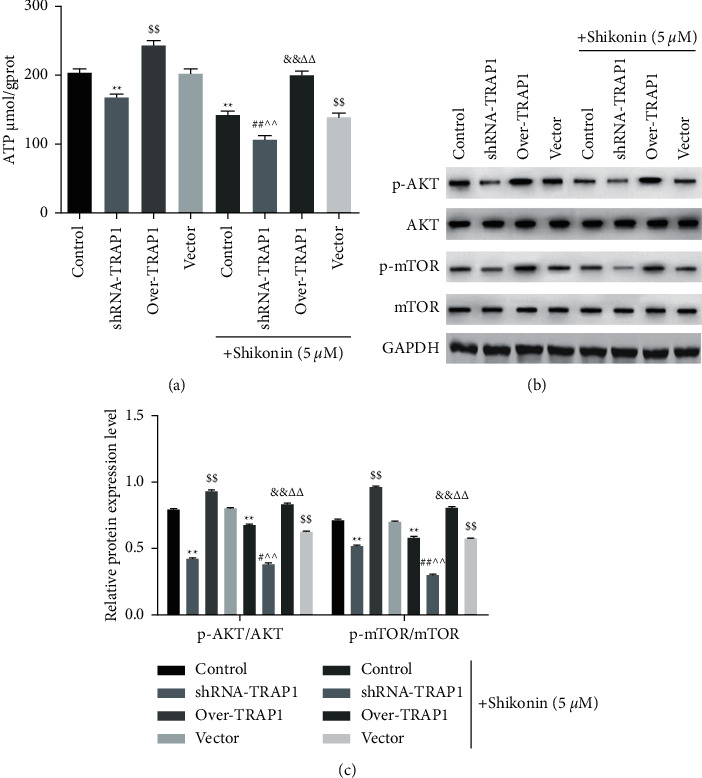
shRNA-TRAP1 enhancing the inhibitory effect of shikonin on the ATP level and AKT/mTOR signaling in TE-1 cells. (a) Comparison of ATP levels. (b) Western blot of p-AKT, AKT, p-mTOR, and mTOR. (c) Quantification of each indicator. Over indicates overexpression.  ^*∗∗*^*P* < 0.01 vs. control; ^$$^*P* < 0.01 vs. vector; ^#^*P* < 0.05. ^##^*P* < 0.01 vs. shRAN-TRAP1; ^&&^*P* < 0.01 vs. over-TRAP1; ^∧∧^*P* < 0.01 vs. shikonin; ^ΔΔ^*P* < 0.01 vs. shikonin + vector.

As shown in Figure 4(a), shRNA-TRAP1 or shikonin inhibited ATP levels compared with that in untreated cells (*P* < 0.01). Compared with the vector group, over-TRAP1 increased the ATP level (*P* < 0.01), while shikonin decreased it (*P* < 0.01). Combined shikonin treatment also increased the ATP level compared with the shRNA-TRAP1 or over-TRAP1 group (*P* < 0.01). The combination of shikonin and shRNA-TRAP1 synergistically decreased ATP release compared with single shikonin treatment (*P* < 0.01), whereas over-TRAP1 increased it (*P* < 0.01). In addition, we examined the activation of the AKT/mTOR signaling pathway during this process (Figures 4(b) and 4(c)). Compared with untreated cells, those treated with shRNA-TRAP1 or shikonin showed decreased the level of p-AKT and p-mTOR (*P* < 0.01). Compared with the vector group, over-TRAP1 increased p-AKT and p-mTOR activity (*P* < 0.01), while shikonin decreased it (*P* < 0.01). Combined shikonin treatment also decreased the level of p-AKT and p-mTOR compared with the shRNA-TRAP1 or over-TRAP1 group (*P* < 0.05, *P* < 0.01). The combination of shikonin and shRNA-TRAP1 synergistically decreased the level of p-AKT and p-mTOR compared with single shikonin treatment (*P* < 0.01), whereas over-TRAP1 showed the opposite effect (*P* < 0.01). These results demonstrated that shRNA-TRAP1 enhanced the inhibitory effect of shikonin on ATP release and AKT/mTOR signaling in TE-1 cells.

## 4. Discussion

An increasing amount of emerging evidence has revealed that shikonin exerts anticancer effects in various cancers. However, whether shikonin exhibits anticancer functions against human EC remains unclear. In the present study, we investigated the suppressive effects of shikonin on EC cells (TE-1) and explored the underlying molecular mechanisms involved therein. We observed that shikonin suppressed EC cell proliferation in a dose-dependent manner and induced apoptosis by regulating apoptosis-related proteins. Flow cytometry uncovered that both shikonin and shRNA-TRAP1 increased the ROS production and mitochondrial depolarization in TE-1 cells while decreasing ATP levels. Furthermore, compared with single shikonin treatment, the inhibition of TRAP1 in TE-1 cells further suppressed cell migration and invasion, which were promoted by the overexpression of TRAP1. More importantly, we confirmed that shikonin induced apoptosis and attenuated human EC cell migration and invasion by inhibiting TRAP1 expression and the AKT/mTOR signaling pathway.

Naturally derived products with anticancer effects have been widely utilized as a source of many medically beneficial drugs. Icaritin, a traditional Chinese herbal medicine that induces sustained extracellular signal-regulated kinase (ERK) 1/2 activation, represses human EC cell growth and promotes apoptosis [18]. In ovarian cancer cells, curcumin induced apoptosis in a dose- and time-dependent manner by inhibiting sarco/endoplasmic reticulum Ca (2^+^) ATPase activity [19]. In human colon cancer cells, luteolin-induced apoptosis was accompanied by the activation of intracellular and mitochondrial ROS scavenging through the activation of antioxidant enzymes [20]. Shikonin has been identified as a potential anticancer agent against human lung cancer [21], prostate cancer [22], breast cancer [23], and EC [24] and showed a significant antitumor effect in EC through regulating hypoxia-inducible factor-1*α*/pyruvate kinase-M2 signaling [24]. Our results demonstrated that shikonin inhibited EC cell growth in a dose-dependent manner and induced apoptosis through activating the intracellular apoptotic signaling pathway. In addition, the downregulation of TRAP1 was synergistic with the antitumor effect of shikonin. These data indicated that shikonin exhibited antiproliferative properties in EC cells and functioned by targeting TRAP1, which could be developed as a potential therapeutic agent against human EC.

As a heterogeneous group of diatomic oxygen from free or nonfree radical species, ROS are derived from mitochondria, which serve as the center of ATP synthesis [25]. Superabundant ROS production reduced antioxidant activity in cells, resulting in membrane lipid peroxidation, mitochondrial dysfunction, and apoptosis [26]. Therefore, a number of therapeutic antitumor approaches have been designed via ROS-mediated mechanisms. For instance, diaporine, a novel small-molecule compound, induced the apoptosis of breast cancer cells by increasing ROS release [27]. The senescence inducer shikonin induced ROS-based mitochondria-mediated apoptosis in colon cancer [28]. In gastric cancer cells, shikonin induced necrosis or apoptosis by generating ROS in a time-dependent manner [29]. In our study, shikonin dramatically induced ROS release. Moreover, downregulation of TRAP1 promoted this effect, whereas upregulation of TRAP1 blocked shikonin-induced ROS release.

Mitochondria are the central executers of apoptosis and are thus the logical targets for apoptosis prevention [30]. Mitochondrial depolarization and loss of mitochondrial membrane potential are characteristics of mitochondrial dysfunction during apoptosis [31]. Because of this, the induction of mitochondrial dysfunction represents another important mechanism involved in the antitumor effect of various drugs. In human papillary thyroid carcinoma cells, shikonin inhibited cell proliferation and led to apoptosis in a dose- and time-dependent manner through mitochondrial pathways, as reflected in enhanced Bax levels, reduced antiapoptotic protein Bcl-2 levels, decreased mitochondrial membrane potential, and activated caspase-3 enzymatic activity [32]. Similarly, shikonin exhibited antitumor activity against HGC-27 human gastric cancer cells through inhibiting cell growth and promoted apoptosis by targeting mitochondrial-related signaling pathways [33]. Our findings demonstrated that shikonin significantly promoted mitochondrial depolarization, which was accompanied by a large release of Cyt-C.

The Warburg effect is one of the metabolic characteristics of cancer cells, whereby, even when oxygen is abundant, cancer cells use glucose through glycolysis and ferment the end product pyruvate into lactic acid rather than completely oxidizing it through the tricarboxylic acid cycle [34]. Although it is unclear why cancer cells shift energy production from the tricarboxylic acid cycle to glycolysis, pyruvate kinase (PK) has been shown to play an important role in this shift. Pyruvate kinase-M2 (PKM2), one of the PK genes, is universally expressed in cancer cells and has vital functions in cancer cell metabolism and growth [35]. Inhibition of PKM2 suppressed cancer cell growth and PKM2 knockdown reduced the tumorigenicity of human cancer cell lines [36, 37]. Hence, PKM2 is a potential molecular target for the disruption of the glucose metabolism in cancer cells. Interestingly, PKM2 was identified as a potential target of shikonin by solid-phase shikonin extraction and mass spectroscopy [38]. In this study, shikonin significantly decreased ATP release, demonstrating its significant intervention in the process of the glucose metabolism.

Generally, most cancers including EC show poor survival and prognosis, partly because of frequent tumor relapse and metastasis. The development of cancer metastasis involves multiple processes, whereby cancer cells detach from the primary tumor, invade surrounding tissues, intravasate into blood and/or lymphatic systems, and extravasate from the vasculature to subsequently settle and colonize at the target organs [39]. MMPs play a key part in tumor metastasis by degrading extracellular matrix proteins [40]. Among them, MMP2 and MMP9 are expressed abundantly in various malignant cancers and degrade type IV collagen. MMP2 and MMP9 may play important roles in cancer cell migration and invasion. For instance, fucoidan treatment at a nontoxic dose (0–200 mg/ml) exhibited a concentration-dependent inhibitory effect on the invasion and migration of cancer cells by suppressing MMP2 activity [41]. In in vitro and in vivo models of osteoarthritis, shikonin inhibited inflammatory responses by reversing the elevated expression of MMP1, MMP3, and MMP13 [42]. Not coincidentally, in ESCC cells, the molecular mechanism of TRAP1-mediated migration and invasion was regulated by the signal transducer and activator of the transcription 3/MMP2 signaling pathway [11]. In the current study, not only shikonin but also shRNA-TRAP1 inhibited TE-1 cell migration and invasion. In addition, shRNA-TRAP1 enhanced the inhibitory effect of shikonin on MMP2 and MMP9 expression.

mTOR is a serine/threonine protein kinase that regulates cell growth, cell motility, cell survival, protein synthesis, and transcription [43]. Dysregulations in the mTOR pathway were observed in human diseases, especially certain cancers [44]. A number of mTOR inhibitors have been introduced in cancer treatment and have demonstrated a good curative effect [45]. AKT is a well-known upstream regulator of mTOR signaling in mammalian cells. As a strong evidence of this concept, siRNA-mediated gene silencing of AKT inhibited the activation of mTOR and subsequently led to the suppression of migration, invasion, and proliferation [46]. In addition, mTOR is a potential target for many anticancer drugs. For instance, cardamomin inhibited the invasion and metastasis of Lewis lung carcinoma cells through inhibiting mTOR activity [47]. In human thyroid cancer cells, metformin inhibited cell growth, migration, and epithelial-to-mesenchymal transition through inhibiting the mTOR pathway [48]. In human prostate cancer cells, shikonin inhibited cell metastasis by reducing MMP2/MMP9 expression via AKT/mTOR and ROS/ERK1/2 pathways [22]. In our findings, we revealed that shRNA-TRAP1 was involved in shikonin-mediated AKT/mTOR signaling regulation and exerted synergistic effects.

## 5. Conclusion

**Figure 5:**
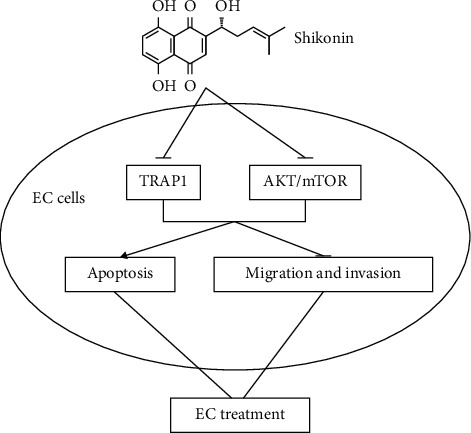
Shikonin promotes apoptosis and attenuates migration and invasion of human EC cells by inhibiting TRAP1 expression and AKT/mTOR signaling.

As shown in Figure 5, our study demonstrated an important role of shikonin in EC cell proliferation and apoptosis and elucidated the molecular mechanism underlying shikonin-mediated migration and invasion. Shikonin promoted apoptosis and attenuates migration and invasion of human EC cells by inhibiting TRAP1 expression and AKT/mTOR signaling, indicating that shikonin may be a new drug for treating EC. However, whether shikonin has the same antitumor effect in vivo is unknown. More fundamental experiments are necessary before shikonin can be applied in clinical trials.

## Data Availability

The data used to support the findings of this study are included within the article.

## Abbreviations

EC: Esophageal cancer
ESCC: Esophageal squamous cell carcinoma
TRAP1: Tumor necrosis factor receptor-associated protein 1
MMP: Matrix metalloproteinase
AKT: Protein kinase B
mTOR: Mammalian target of rapamycin
Cyt-C: Cytochrome C
BCA: Bicinchoninic acid
CCK-8: Cell counting kit-8
FBS: Fetal bovine serum
GAPDH: Glyceraldehyde-3-phosphate dehydrogenase
ROS: Reactive oxygen species
ATP: Adenosine 5′-triphosphate
PBS: Phosphate-buffered saline
Over-TRAP1: Overexpression of TRAP1
PKM2: Pyruvate kinase-M2.
